# Circadian Rhythms and Hormonal Homeostasis: Pathophysiological Implications

**DOI:** 10.3390/biology6010010

**Published:** 2017-02-04

**Authors:** Davide Gnocchi, Giovannella Bruscalupi

**Affiliations:** 1 Department of Laboratory Medicine, Division of Clinical Chemistry, Karolinska Institutet at Karolinska University Hospital Huddinge, Stockholm 14186, Sweden; 2 Department of Biology and Biotechnology “Charles Darwin”, Sapienza University of Rome, Rome 00185, Italy; giovannella.bruscalupi@uniroma1.it

**Keywords:** circadian clock, central oscillator, peripheral oscillators, hormonal homeostasis, metabolic diseases

## Abstract

Over recent years, a deeper comprehension of the molecular mechanisms that control biological clocks and circadian rhythms has been achieved. In fact, many studies have contributed to unravelling the importance of the molecular clock for the regulation of our physiology, including hormonal and metabolic homeostasis. Here we will review the structure, organisation and molecular machinery that make our circadian clock work, and its relevance for the proper functioning of physiological processes. We will also describe the interconnections between circadian rhythms and endocrine homeostasis, as well as the underlying consequences that circadian dysregulations might have in the development of several pathologic affections. Finally, we will discuss how a better knowledge of such relationships might prove helpful in designing new therapeutic approaches for endocrine and metabolic diseases.

## 1. Introduction

The idea of the existence in higher organisms of internal rhythms that oscillate autonomously, synchronising and regulating many biological functions was not easy to accept by the scientific community. The study of biological rhythms was in fact initially considered with diffidence, and the concept of an internal self-governing timekeeper, coordinated only by external stimuli such as light, was difficult to acknowledge. Nonetheless, this topic is now being investigated by an increasing number of researchers.

Hormonal homeostasis exhibits periodic fluctuations; it is now becoming clear that endocrine rhythms and circadian rhythms are tightly interconnected, and that the internal clock deeply interacts with environmental factors to maintain the internal balance.

Here we aimed to review the importance of circadian rhythms, describing how they were discovered and how their organisation from an anatomical and molecular point of view was brought to light. Then we will concentrate on the association between circadian clock and hormonal homeostasis, focusing on the pathophysiological consequences of circadian clock desynchronisation on endocrine balance and hence on our health.

## 2. Circadian Rhythms

### 2.1. Historical Background

The existence in higher eukaryotes of an internal periodicity, independent of environmental stimuli was first documented by Jean-Jacques d’Ortous De Mairan at the beginning of the 18th century. In the leaves of the plant *Mimosa pudica*, he observed a 24-h periodicity in the opening–closing cycles that was retained when the plant was kept in the dark. In the middle of 19th century, Alphonse de Candolle, in the same plant, noticed that the length of the leaf cycle was not precisely 24 h. In the middle of the 20th century, Erwin Bünning reported that the leaf cycle of *Phaseolus coccineus* presented an average periodicity of 24.4 h [1]. In the same years, Colin Pittendrigh and his collaborators found that likewise, in the fruit fly *Drosophila*, the daily cycle oscillated between 22 and 28 h [2,3,4]. These findings introduced a key concept for the understanding of circadian rhythms: the periodicity is not provoked by external stimuli, but is “innate”, the average period being about 24 h. Such internal autonomous oscillations were termed “free-running” rhythms, and external environmental stimuli are needed to synchronise them. Light is the main “timer” (“Zeitgeber” in German) that regulates the free-running cycle and synchronises (“entrain”) mammalian internal clocks with the environmental time. Light modulates retinal inputs at the central nervous system (CNS) level. Such light entrainment necessitates neither rods nor cones, since retinal ganglion cells innervating the CNS are intrinsically photosensitive, and depolarise in response to light also in the absence of all synaptic inputs from rods and cones [5]. Since the sensitivity, spectral tuning and kinetics of the response to the light fit with those of the photic entrainment, these ganglion cells are now believed to be the primary photoreceptors for this system [5].

### 2.2. The “Molecular” Clock

Since those first discoveries, this field of research has significantly developed, and now many physiological and biochemical aspects of our internal clocks have been clarified.

The experiments that led to the discovery of the localisation of the internal oscillator and to the clarification of the nature of the physiological mechanisms involved, were performed in mammals. The first step was determining that rats with lesions in the hypothalamus lost most of their behavioural periodicity [6,7]. The actual anatomical site of the clock was subsequently associated in rats to the suprachiasmatic nucleus (SCN), a small group of cells lying in the anterior hypothalamus [8]. The neural connection linking the eye to the SCN was also elucidated in rats, and termed the “retinohypothalamic tract” (RHT) [9]. Employing rats and golden hamsters, the regulation of internal periodic behavioural patterns was demonstrated to be an intrinsic property of the SCN, which produces a periodic rhythmic electrical activity with a rate of around 24 h. Such an electrical oscillation in turn produces an analogous pattern in the neighbour neurons, but with a phase shift—in nocturnal animals mainly during the night [10,11,12].

The molecular characteristics of the circadian clock were identified as a result of a series of experiments performed employing mutants of *Drosophila* and mice. The first gene discovered was called *period (Per)*, since the mutant flies had an altered eclosion time [13]; the protein codified by this gene (PER), showed rhythmic cycles of synthesis that were well synchronised with the circadian behaviour of the fly [14]. A complex negative feedback loop model explaining those observations was suggested after the individuation of three additional proteins: (a) the product of the *timeless (Tim)* gene, TIM, discovered in *Drosophila* [15,16]; (b) CLOCK, whose gene *clock* was individuated in mice [17]; and (c) CYCLE (CYC) whose gene *cycle* was described in *Drosophila* [18]. In *Drosophila*, the regulatory mechanism works through the formation of a CLOCK·CYCLE complex, which binds to the E-box region on *Per* and *Tim* promoters, thus stimulating their transcription. When PER and TIM level increases, the PER·TIM complex interacts with CLOCK·CYCLE, stopping its function as a transcriptional activator. When the transcription is blocked, a new cycle of synthesis can begin. This mechanism is further fine-tuned by the doubletime (DBT) protein kinase, which phosphorylates PER, addressing it to proteasomal degradation. DBT is able to phosphorylate PER only when it is in its free form, but not when it is found complexed with TIM: for such a reason the PER·TIM complex forms only when there is an increase in the level of TIM, which inside the nucleus is stable for about 10 h. The light entrainment is achieved by two different light-catching systems, which work through two distinct photopigments: cryptochrome (CRY), and opsin. After the light has been sensed, the stimulus leads to TIM degradation, which in turn will reset the whole mechanism [19].

The clock of mice differs in some key aspects if compared with the *Drosophila* clock. Three *Per* homologue genes have been identified so far, namely *mPer1–3*. Their transcription is quickly activated by light, with a period of nearly 24 h [20,21,22]. So far, no evidence supporting the presence of a gene performing a function equivalent to that of *Tim* has been found in *Drosophila*, even if an essential role for one of these possible analogues in entraining the clock in cooperation with *Per1/2* was suggested [23]. Several lines of evidence demonstrate the involvement of *Tim* in the regulation of developmental processes, hence substantiating the hypothesis of an important role played by the circadian clock in the early developmental stages in mammals. In fact, in mouse and rat embryos, *Tim* is highly expressed in the developing lung, liver, and kidney, as well as in neuroepithelium, supporting its role in epithelial organogenesis [24]. Furthermore, *Tim* regulates the apoptotic processes involved in mouse embryonic stem cell differentiation [25].

The mammalian analogue of *cycle* was termed *Bmal1* [26]. In mammals, *Cry* has two isoforms, *Cry1* and *Cry2*. Even if not playing a direct role in the reception of light, *Cry* is necessary for the feedback mechanism [27,28,29]. Additional players, namely *Rev-Erb α/β* and *ROR α/β*, further refine the mechanism. They interact with the RORE enhancer element in the *Bmal1* gene, and repress and stimulate its transcription respectively [30,31,32,33]. Although formerly considered not fundamental for the generation of circadian rhythmicity, they are now acknowledged to play a significant function [34]. Finally, the mammalian functional analogues of *Drosophila* DBT are casein kinase 1ε (CK1ε) and casein kinase 1δ (CK1δ), which, as a complex (CK1δ/ε), phosphorylate PER proteins, directing them to proteasomal degradation [35,36]. Therefore, in mammals the overall process commences after the light is detected by both “classical” photoreceptors, and by the photoreceptor melanopsin, which is located in the retinal ganglion cells that form the RHT tract and project to the SCN [5,37,38]. Light then activates a chain of responses that in turn reset the clock by increasing *mPer1-2* transcription [39,40,41]. The de novo synthesised PER proteins, after binding to CRY, enter into the nucleus, where the PER·CRY complex inhibits the transcription of clock controlled genes (ccg) and of their own genes, whose expression is typically activated upon the binding of CLOCK·BMAL1 to the E-box enhancer elements [42,43].

As described above, an additional checkpoint is represented by the CK1δ/ε-mediated PER·CRY phosphorylation, which addresses the complex to proteasomal degradation. It has been proposed that CLOCK may own histone acetyltransferase (HAT) activity, which increases after the binding of its heterodimeric partner BMAL1. This HAT activity of CLOCK is fundamental to restore circadian rhythmicity and the activation of circadian genes in *Clock* mutant cells [43]. In mouse liver, CLOCK was also shown to acetylate BMAL1, which underwent rhythmic acetylation with a timing that parallels the down-regulation of circadian clock-controlled gene transcription. BMAL1 acetylation facilitates CRY1 recruitment to CLOCK-BMAL1, thus triggering transcriptional repression. Consequently, this enzymatic interaction between the two clock components is central for the proper functioning of the circadian mechanism [42,43].

The circadian electric activity produced in the SCN has to be transmitted to the rest of the brain and then be translated in signals that might be received by the peripheral districts of the body. The SCN transmits signals in three main ways: (a) neuronal networking, by directly taking contact with several other brain regions; (b) chemically, by synthesising signalling molecules; and (c) indirectly, by setting rest-activity rhythms that in turn trigger feeding-fasting cycles, which were shown to represent the principal Zeitgeber for the synchronisation of the clock of several peripheral organs [44,45]. In fact, the SCN interacts directly with the subparaventricular zone (sPVZ) [46,47], the preoptic area (POA), the bed nucleus of the stria terminalis (BNST), the lateral septum (LS), the dorsomedial hypothalamus (DMH), the arcuate nucleus (ARC), and the paraventricular nucleus (PVN) [46,47]. The signal is then conveyed from the SCN to the above-mentioned structures through classical neurotransmission mediated by GABA and glutamate [48]. The SCN also produces several signal molecules, which in turn act on adjacent structures. Amongst the best characterised so far are arginine vasopressin (AVP) [49,50,51], vasoactive intestinal peptide (VIP) [48], cardiotrophin-like cytokine [52], prokineticin 2 (PK2) [53], epidermal growth factor (EGF) [54] and transforming growth factor α (TGFα) [55].

### 2.3. Peripheral Clocks

The molecular machinery that controls the circadian activity is expressed not only in the SCN, but also in almost all the peripheral tissues, as at first shown in rodents [56]. Such peripheral rhythms are however reliant on the activity of the central pacer, since in vitro they attenuated until blocking after 2–7 cycles in the liver, lung and skeletal muscle, in the absence of the contribution of the SCN [56]. Following research described that peripheral tissues in isolation were able to self-sustain a circadian periodicity for more than 20 cycles. Moreover, peripheral organs showed tissue-specific differences in circadian period and phase, and lesions of the SCN did not reset circadian rhythmicity, but just desynchronized peripheral tissues of individual animals and from different animals. Therefore, peripheral organs express at least a partially self-sustained circadian oscillator [57]. Such circadian behaviour was observed also in vitro in several cell types [58,59,60,61], and in tissue explants from almost all the organs [57,62]. Interestingly, the brain itself possesses its own circadian oscillation in the expression of several genes that seems to be independent of the activity of the SCN, at least according to what was shown in the olfactory bulb [63,64].

A comprehensive bioinformatics study performed in mice led to the identification of 41 circadian genes that oscillated in a circadian way in several mouse tissues with a considerable consistency of circadian phases in the different tissues. Interestingly, comparisons between mouse, rat, rhesus macaque, and man revealed that the phase of key circadian genes presented a delay in the other species if compared to mice (4–5 h in rats, 8–12 h in macaques and humans). Overall, approximately 2%–10% of the entire genome displayed a circadian pattern of expression in several tissues. Part of such genes was expressed tissue-specifically; nevertheless, almost all tissues shared the vast majority of them. The genes involved in the periodicity—such as *Per2*, *Bmal1*, *Rev-erbα* and *Cry*—presented the higher degree of conservation [65]. On the other hand, from a study that compared the oscillating transcripts in mouse liver and NIH3T3 and U2OS cells, it emerged that the number of cycling transcripts in cellular systems was very different if compared to tissues from intact mice. In particular, two big gene clusters cycled in the liver, but not in cultured cells. Interestingly, a 12-h oscillatory transcript rhythm was observed also in other peripheral tissues, including the heart, kidney, and lung. Such patterns were lost ex vivo and under restricted feeding conditions. This study clearly demonstrated the presence of circadian harmonic of gene expression in mice, while recommending prudence when approaching to the study of circadian clock employing cellular models [66].

#### 2.3.1. Entrainment of Peripheral Clocks

Three main sources of entrainment are involved in the synchronisation of peripheral clocks: (a) direct entrainment by the SCN through neural and hormonal signals; (b) entrainment through feeding-fasting rhythms; (c) body temperature entrainment.

Neural control is undertaken through the autonomic nervous system, whose outputs are in turn indirectly controlled by the SCN. The SCN seems to have a multifaceted role in the entrainment of the different peripheral organs. The extent of the involvement of the SCN in this regulation and which mechanisms may possibly be involved is still under investigation. It is well-known that damages in the SCN result in the elimination of circadian patterns of feeding and drinking [8]. In a study performed in mouse liver, about 9% out of around 2000 genes screened showed an evident circadian cycling pattern. The circadian regulation of these genes was tissue specific, since the new-identified rhythmic hepatic genes did not show a rhythmic expression in the brain, even when detected in the SCN. Thus, since SCN ablation severely compromised cyclical expression of liver circadian genes, authors concluded that the circadian rhythmic transcriptome in peripheral organs is strictly SCN-dependent, but for proper functioning it needs a crosstalk between tissue-specific factors and SCN regulation [67]. In SCN-deficient rats, light did not trigger the sympathetic-induced corticosterone release by the adrenal gland [68]. Likewise, in rats without the SCN, the hyperglycaemic effect of GABA antagonists was lost [69]. In rats whose autonomic liver innervation was surgically removed, light could not induce *Per1*/*2*, *Pepck* and *Glut2* up regulation [70]. Interestingly, in rats, fast/feeding schedule and light cycles contributed to set the phase of clock genes in submaxillary salivary gland. Also, after SCN sympathetic denervation, *Per*1 rhythms in submaxillary glands shifted their phase and entrained to daytime feeding. Hence, authors suggest that peripheral oscillator entrainment may be achieved through the synergistic action of diverse signals and that the elimination of the dominant SCN signal may leave the control to a secondary signal [71].

Feeding-fasting rhythms exert a central role for the entrainment of several peripheral organs, such as the heart, kidney, pancreas and liver. In mouse liver, the vast majority of the genes that are involved in the regulation of metabolic pathways are expressed in a circadian way [72]. In rats which feed during the night, the inversion of feeding rhythms—namely the artificial induction of a diurnal eating—promptly and considerably altered the expression of metabolic genes in the liver; the rhythmicity was slightly affected also in the lung [73]. In *Cry1/2*-deficient mice, temporally restricted feeding restored the circadian transcriptional periodicity of the vast majority of the hepatic genes. In contrast, in the absence of a fixed feeding schedule, the animals maintained the transcriptional periodicity of just the minor part of the usual circadian-expressed genes [45]. The regulation of circadian rhythms in peripheral tissues by feeding/fasting is achieved also by hormones such as peptide YY, oxyntomodulin, cholecystokinin, leptin, and ghrelin, which directly signal to the arcuate nucleus (reviewed in [74]).

The third important factor for circadian clock regulation in peripheral tissues is temperature, even if the mechanisms involved are so far not completely clarified. In mice the SCN is able to compensate for temperature variations of the external and internal environment; this process seems to be mediated by CRY and PER. Also, heat-shock factor 1 (HSF1) inhibition mimicked the effect of cool pulses, while blocking HSF1 induction resulted in a loss of resetting after warm pulses. Hence, authors suggested a key role of HSF1 pathway in temperature entrainment in mammals [75]. Likewise, it was reported that in cultured fibroblasts from both mice and humans, simulated body temperature cycles, with daily temperature differences of 3 °C and 1 °C respectively, were able to synchronize circadian gene expression. After few days, gene expression was actually synchronized with temperature cycles, and such temperature rhythms also entrained gene expression cycles to periods longer or shorter than 24 h. Interestingly, HSF1 but not HSF2, was needed for fibroblast oscillator synchronization to simulated body temperature cycles. Thus, the authors suggested a model according to which in peripheral cell types elevated temperatures induce HSF1 activity, which, in cooperation with other temperature-sensitive regulators, promote the expression of immediate early genes (IEGs) such as *Per2*. In turn, IEGs may phase-reset other clock genes such as *Bmal1*. Authors also do not exclude a direct action of temperature on clock protein activity and stability [76]. Thus, working synergistically to orchestrate the circadian phase, light and temperature modulate the regulation of the clock, contributing to the seasonal adaptations of clock functions.

#### 2.3.2. Liver and Pancreas Clocks

The level of expression of many hepatic genes, both clock-related and hepatic-specific, follows a circadian periodicity. Circadian genes play a complex role for liver functions. *Bmal1*-deficient mice lost the rhythmic behaviour in both the brain and the liver, while *Clock*-deficient mice showed a not functional hepatic circadian clock but preserved central periodicity [77]. Nonetheless, liver periodicity shows a relative autonomy from the central regulator. In mice, the glucocorticoid receptor rescued about 60% of circadian gene expression that was lost by damaging the SCN [78]. Food also plays a key role, even when the central pacemaker is correctly working, since more than 80% of the hepatic transcriptome is “meal-dependent” [73].

The circadian oscillator plays a fundamental role in the regulation of glucose metabolism. In *Bmal1*- and *Per1/2*-deficient mice, liver-specific *Bmal1* inactivation resulted in severe hypoglycaemia during the inactivity period, but not if *Bmal1* was inactivated in the other entire cell types excluding the liver [77]. A recent study showed that *Per2* controls glucose homeostasis in humans as well [79].

In mice, bile acid and cholesterol biosynthesis regulation is under circadian control through *Rev-Erbα*, which governs SREBP expression, and thus that of cholesterol metabolism genes. It was also proposed that the cyclic expression of *cholesterol-7α-hydroxylase* (*Cyp7a1*) might be driven by a REV-ERBα-mediated mechanism, by means of an oxysterol-mediated LXR activation [80].

The existence of an independent circadian oscillator in the pancreas was recently demonstrated. *Bmal1* and *Clock* play a key role, since their selective knockdown in mice led to modifications of the proliferative rate and size of islet cells, together with hypoinsulinaemia, reduced glucose tolerance and diabetes [81,82].

## 3. Circadian Clock in Hormonal Homeostasis

Several hormones were shown to have daily oscillations, and among these the best characterised are melatonin, cortisol, gonadal steroids, prolactin, thyroid hormone and growth hormone (GH). The so-called nutrient-sensitive hormones, namely insulin, leptin, ghrelin and adiponectin also oscillate on a circadian basis, and their release is, at least in part, regulated by environmental stimuli, such as feeding time and light–dark cycles.

### 3.1. SCN as a Controller of Endocrine Homeostasis

#### 3.1.1. Melatonin

SCN directly interacts with the pineal gland through the sympathetic neurons of the superior cervical ganglion [83]; in turn, the rhythmic activity of the SCN determines the release of melatonin, which directly correlates with day length. In both nocturnal and diurnal animals, melatonin production peaks in the middle of the night, between 24:00 and 03:00, inducing activity in the former and rest/sleep in the latter.

Melatonin plays several key roles, and can be considered the central “relayer” which conveys information about light–dark cycles. In mammals, melatonin is also essential in the regulation of reproductive behaviour and sleep.

Melatonin functions as a feedback regulator on SCN. Melatonin receptors MT1 and MT2 are expressed at high densities in SCN [84]. In rats kept in the dark and in blind humans, melatonin entrained the free-running rhythm [85,86,87,88,89,90]. in vitro, melatonin also regulated the phase and amplitude of the electric circadian activity of SCN explants [91,92]. MT2 receptor was shown to mediate the phase-synchronising effect of melatonin on SCN [93].

Interestingly, in both primates and humans, melatonin modulates adrenal glucocorticoid production, being able to suppress cortisol production [94,95,96]. This effect is mediated by MT1 receptor [96]. Such effects were also observed in foetal rats, where melatonin entrained adrenal gland secretion rhythms [97]. The secretion of several hormones, namely gonadotropin-releasing hormone (GnRH), luteinising hormone (LH), and follicle-stimulating hormone (FSH) is likewise under melatonin control [98]. Melatonin is also synthesized in peripheral tissues, such as the gastrointestinal tract, the retina, skin, lymphocytes and bone marrow, from which it may in turn modulate other physiological functions through paracrine signalling.

After its discovery, many hypotheses have been proposed about the factual role of melatonin for the regulation of biological functions [99]. In fact, melatonin was shown to affect several physiological functions, such as blood pressure regulation [100], immune system modulation and free radical scavenging [98]. In addition to its well-known role as a circadian clock pacer, melatonin was also suggested to positively affect mood disorders as well as cardiovascular, gastrointestinal and bone physiology. Also, melatonin was addressed in playing a role as an oncostatic molecule [98], even if more clinical trials will be necessary to ascertain its possible future role in tumour therapy [101]. In fact, some reports underlined that the evidence gathered so far is inconclusive and not sufficient to corroborate such a view [102,103].

#### 3.1.2. Vasopressin, Acetylcholine, Adrenocorticotropic Hormone

The circadian activity of the SCN directly influences the rhythmic secretion of several other hormones. Arginine vasopressin (AVP), or simply vasopressin, is produced in paraventricular (PVN) and supraoptic (SON) nuclei of the hypothalamus and transported to the posterior pituitary, whence it is released in the system circulation, reducing water elimination from kidneys to prevent dehydration. The SCN directly regulates AVP secretion into the cerebrospinal fluid; as a SCN neurotransmitter, AVP is also essential as an autocrine regulator and pacer of the neuronal activity [50,104].

The neurotransmitter acetylcholine (ACh) was one of the first neurotransmitters suggested to be involved in circadian rhythmicity and actually, it shows a circadian pattern of release, which is high during the active phase [105,106]. Despite of this, the evidence collected so far is not conclusive. The cholinergic system in mammals (men included) shows a marked circadian activity. ACh is released during wakefulness and motor activity, whereas a reduced release is observed during sleep. It was hence speculated that behavioural activity patterns, circadian rhythms, and cholinergic neurotransmission were tightly coupled. Several studies have indeed demonstrated a role of cholinergic signalling in the regulation and maintenance of circadian rhythms via nicotinic and muscarinic acetylcholine receptors (nAChRs and mAChRs, respectively) [106]. In any case, further investigations will be necessary to more deeply clarify the role of ACh in the regulation of circadian rhythms.

The SCN coordinates also the periodic release of glucocorticoids from the adrenal cortex [107], which results in the maximal production in the early morning for diurnal animals, and in the early evening for nocturnal ones [108,109]. Adrenocorticotropic hormone (ACTH), which induces the release of corticosterone from the adrenal cortex, shows a similar pattern of release from the corticotrope cells of the pituitary [108,109,110]. Such a process is repressed by light, and it seems to be directly dependent on SCN through its connections to the paraventricular nucleus [108,109,110]. Such precise control is crucial considering the key functions performed by this hormone, both as a precursor of aldosterone and for the regulation of hepatic metabolism [111]. Of note, the glucocorticoid receptor agonist dexamethasone synchronises circadian gene expression in rat fibroblasts and shifts the phase of expression of circadian genes in the liver, kidney, and heart. Due to the lack of glucocorticoid receptors in the SCN, dexamethasone does not modify its circadian behaviour [78,112].

#### 3.1.3. Cortisol

Among all glucocorticoid hormones, cortisol is one of the best characterised from a circadian point of view. In humans, cortisol production usually increases during the night and shows a peak of secretion in the morning, around 07:00–08:00, in this way setting the endocrine balance for the stress associated with waking [113,114].

Jet lag and sleep desynchronisation were shown to increase cortisol levels in humans [115,116], and increased cortisol was associated with several pathologies such as cardiometabolic diseases, sleep and mood disorders, [117] and tumours [118,119].

Glucocorticoids and cortisol can modulate the expression of clock-controlled genes in the liver, kidney and adipose tissues [120,121]. Moreover, in a mouse model of jet lag, glucocorticoids were identified as key modulators for clock resynchronisation [122]. Interestingly, it was recently reported that in rats, clock genes showed their usual circadian rhythms after adrenalectomy and feeding/fasting rhythm disruption, even if the hepatic neuronal inputs were maintained. These data underline the importance of fasting/feeding cycles and of adrenal hormones for a proper synchronization of the hepatic clock with the SCN [123].

#### 3.1.4. Insulin and Ghrelin

Insulin and ghrelin represent two key factors in metabolic regulation, and now several circadian factors are recognised to regulate their secretion and activity.

In humans, insulin secretion shows a zenith at around 17:00 and a nadir at about 04:00, thus promoting nutrient storage in the active phase. The clock tightly controls insulin secretion, since deficiency in both CLOCK and BMAL1 determines hypoinsulinaemia [81,82], whereas loss of PER and CRY causes hyperinsulinaemia [124,125]. Shift work was shown to determine a rise in insulin secretion together with a decrease in insulin sensitivity, possibly implying a pre-diabetic condition [126,127]. Insulin and glucose are also able to control the clock. In a three-dimensional rat hepatocyte model and in primary mouse hepatocytes, insulin resynchronised the liver clock [128,129]. Moreover, glucose was shown to down-regulate *Per1* and *Per2* expression in cultured rat fibroblasts [130].

The oxyntic cells of the stomach secrete ghrelin before feeding time, independently from light, according to their own circadian clock [131]. The main action of ghrelin is appetite stimulation [132,133,134]. It was shown that in shift workers the normal ghrelin cycle was disrupted, possibly explaining the observed overfeeding [135]. In mice, ghrelin also directly regulated the expression of clock genes in the SCN, increasing food intake [136].

#### 3.1.5. Adiponectin and Leptin

Adiponectin is a so-called adipokine, since it is secreted by adipose tissue. In humans, its peak of production is observed between 12:00 and 14:00 [137,138]. Adiponectin is known as an anti-inflammatory and insulin-sensitizer molecule [139]; its level was inversely correlated with obesity, and decreased weight results in its increase [140,141]. In rodents under high-fat diet (HFD), an inverse correlation was demonstrated between fat mass and adiponectin levels [142,143]. In a mouse model of metabolic syndrome with hypoadiponectinemia, animals showed a reduced circadian locomotor activity, but an increased activity during the light-phase. Also, circadian gene expression was shifted in the liver and skeletal muscle. Restoring the adiponectin gene expression in the liver resulted in the recovery of the correct locomotor activity pattern, together with hepatic clock gene expression [144].

Leptin is secreted by the white adipose tissue after the hepatic glucose level increases, and acting at the level of the appetite centres in the hypothalamus, it conveys signals of satiety, preventing overfeeding. In humans, leptin levels peak during the night. In humans, HFD lowers leptin levels, while increased fat mass and obesity results in hyperleptinaemia [145]. In female mice, leptin increased *Per* expression in the SCN, increasing light phase-shifting action [146]. In rats, ex vivo, leptin was shown to reset SCN clock phase [147].

## 4. Circadian Clock and Metabolism Regulation

In the last two decades the comprehension of the influence of circadian clock on metabolism regulation has greatly improved [148,149,150].

A central role was attributed to SIRT1, a member of SIRT deacetylase family, whose activation has been related with many positive effects [151]. SIRT1 activity requires the presence of NAD^+^ as a cofactor, consequently, during fasting, when NAD^+^ level is elevated, SIRT1 activity is high [151]. SIRT1 modulates the rhythmic expression of numerous circadian controlled genes. NAD^+^-dependent histone deacetylation mediated by SIRT1 of BMAL1 and PER2 enabled the establishment of a repressive chromatin state [152]. SIRT1 binds with CLOCK and is recruited at the CLOCK·BMAL1 chromatin complex at circadian promoters. Genetic disruption of *Sirt1* or pharmacological inhibition of SIRT1 desynchronised the circadian cycle: so SIRT1 might play a role as a controller of the circadian machinery, perceiving modifications in cellular metabolite level [153]. Of note, intracellular NAD+ levels exhibited circadian oscillations, as a consequence of the circadian expression of nicotinamide phosphoribosyltransferase (NAMPT) mediated by CLOCK·BMAL1. SIRT1 is then recruited to the *Nampt* promoter, directing the synthesis of its own coenzyme [154]. SIRT1 is also involved in the regulation of circadian transcription of numerous clock genes, namely *Bmal1*, *Per2*, *Cry1*, and *Rorγ*. SIRT1 binds CLOCK·BMAL1 and promotes PER2 deacetylation and degradation. Because its deacetylase activity is dependent upon NAD^+^ levels, SIRT1 may function as a connector between cellular metabolism and the circadian machinery [155]. Interestingly, it was shown that SIRT1 stimulates *Bmal1* and *Clock* transcription in the brain, by activating a positive feedback loop involving SIRT1, PGC-1α, and NAMPT. Aged mice showed decreased SIRT1, BMAL1 and PER2 levels in the SCN, resulting in a deregulated activity pattern and light entrainment. These effects were not observed in SCN SIRT1-overexpressing mice [156].

AMPK is a multi-protein complex that plays a central role in metabolism regulation as a general stimulator of catabolic pathways and inhibitor of anabolic ones. One of its regulatory subunits, *ampkβ2*, is expressed in a circadian way, which results in a periodic translocation into the nucleus, where it directly phosphorylates CRY1 in association with LKB kinase. This phosphorylation determines CRY1 proteasomal degradation [157].

BMAL1 and CLOCK are very important for glucose and triglyceride homeostasis regulation. Gluconeogenesis was in fact stopped by *Bmal1* deletion and reduced in *Clock* mutants. Furthermore, high-fat diet amplified circadian oscillations in insulin sensitivity and glucose tolerance [158]. The circadian clock also supervises hepatic gluconeogenesis, which during fasting is started by the cAMP-mediated phosphorylation of cAMP response element-binding protein (CREB). CREB activity is regulated during fasting by *Cry1* and *Cry2*. *Cry1* expression is high during the night–day transition, when it reduces fasting gluconeogenic gene expression by inhibiting glucagon-mediated increase in intracellular cAMP concentrations and protein kinase A-mediated phosphorylation of CREB. As hepatic overexpression of *Cry1* lowers blood glucose concentrations and improves insulin sensitivity in insulin-resistant mice, molecular cryptochrome activity enhancers might be considered as useful therapeutic agents for type 2 diabetes [159].

The association between circadian clock and metabolism was further substantiated by the discovery that the highly metabolically controlled transcription factor REV-ERBα is central for circadian clock synchronisation [160]. Synthetic REV-ERBα agonists were in fact suggested as positive regulators for metabolic diseases. In mice treated with synthetic REV-ERB agonists, the circadian expression of metabolic genes in the liver, skeletal muscle and adipose tissue was modified, leading to augmented energy expenditure. In fact, diet-induced obese mice treated with REV-ERB agonists showed decreased fat mass and improved hyperglycaemia and dyslipidaemia [161].

Additional points of contact between circadian clock and metabolism, and in particular with the development of metabolic diseases were found. For example, the activity of the stomach ghrelin-secreting cells is synchronised by food through a clock-driven mechanism [131]. Of note, glycogen synthase kinase-3 (GSK-3β) inhibition resulted in a period shortening of the clock [162].

## 5. Health Consequences of Circadian Misalignment

Several pathologic affections have been associated with deregulations and disruptions of circadian rhythms.

In rodent models, experimental jet lag approaches with long-term light-dark shifts, resulted in reduced body temperature, increased adiposity, altered immune response and tumour development [163,164,165,166]. Forced desynchronisation of light–dark cycles also resulted in perturbed hormonal homeostasis [122,167,168].

In humans, induced sleep–wake misalignment caused an unscheduled secretion of insulin, leptin and norepinephrine, whereas cortisol, epinephrine and glucose kept a normal circadian secretion pattern [169]. Circadian misalignment induced by sleep deprivation increased markers of insulin resistance and inflammation [170].

## 6. Possible Circadian-Based Therapeutic Approaches

It is now clear how much our biological clock is deeply interconnected with our endocrine homeostasis, and how perturbations of our hormonal balance can result in pathologic consequences. Hence, finding new therapeutic approaches to cope with the increased incidence of circadian-related affections might prove essential in the next years.

Many genetic approaches have been suggested which involve direct actions on clock genes, together with behavioural techniques aiming to change wrong eating and sleeping attitudes. It is anyway easy to understand that such kinds of approaches are difficult to be performed. In this light, the development of new molecules targeting clock protein might prove useful. So far, several agents have been discovered that may work in this sense:
KL001.This molecule stabilises CLOCK protein in vitro, in this way increasing the length of the clock period. KL001 also decreases hepatic glucose production [171].SR9009 and SR9011.These molecules are REV-ERBα agonists, and were shown to decrease *Cry2* rhythms, to increase *Per2* rhythms and to shift *Bmal1* ones. SR009 was also effective in decreasing adiposity and in improving dyslipidaemia and hyperglycaemia in a mouse obesity model [161].Longdaysin.This molecule was shown to increase circadian period both in vitro and *in vivo* by inducing PER1 degradation through the modulation of several kinases, and in particular CKIα [172].

Finally, it is worth mentioning two approaches that are becoming quite popular for the treatment of circadian and circadian-related disorders: light therapy and chronotherapy.

In the last years a large part of the general population has modified the sleeping schedule during the weekends in comparison to working days, leading to the so-called “social jetlag”, which was associated with negative consequences for health. Light therapy in the morning was suggested as the most effective approach to advance the circadian rhythm and sleep phase. In a recent study it was demonstrated that short (30 min) blue light pulses in the morning restored sleep integrity and improved performance impairment. Interestingly, the effect was not observed when using the amber light [173]. Another very recent report demonstrated that in a small human clinical trial, intermittent light was more effective than continuous light in shifting circadian phase. Authors showed that there is an optimal interstimulus interval between the flashes where light flashes were around 2-fold more effective in phase delaying the circadian system when compared to continuous light exposure about 4000 times longer the duration of the most effective stimulus. Light pulses did not affect melatonin levels or alertness [174].

Chronotherapy may be defined as the approach employed to maximise the efficacy and minimise the side effects of a drug treatment by administering it taking into account circadian rhythms. So far, chronotherapy has been considered and tested for several different types of diseases, such as hypertension [175,176,177,178,179], rheumatoid arthritis [180], inflammatory diseases [181,182] depression [183] and tumours [184,185]. Even if convincing evidence has been reported with respect to the efficacy of such approaches, more clinical trials will be necessary to fully unravel their actual biological and therapeutic practical relevance.

## 7. Conclusions

Circadian rhythms are now acknowledged as vital constituents of the multifaceted physiological machinery that controls and regulates many essential physiological mechanisms in most organisms, from cyanobacteria to mammals. The strong association between the circadian clock, hormonal homeostasis and energy metabolism has been brought to light during the last two decades, showing the role of the central clock in the coordination of the clocks in the peripheral organs. So far, numerous genes involved in metabolism control have been discovered to be under circadian regulation; therefore, any modification of this delicate equilibrium often results in the development of severe pathological conditions. Several points are, in any case, still under investigation, and potential new breakthroughs will be beneficial not only to increase scientific knowledge, but also to improve public health.

In fact, in the last years, an increasing portion of the general population modified the lifestyle often resulting in a decrease and a worse quality of the sleep. Shift work and/or frequent transmeridian flights have also become more common. Circadian rhythm disruption was in fact associated to an increased incidence of metabolic, endocrine, cardiovascular, and tumour diseases. It is, in any case, difficult to ascertain the actual relative contribution of circadian disruption to the onset of such diseases, since bad habits, such as smoking, alcohol consumption or reduced physical activity might have arisen as secondary consequences of shift work or frequent transmeridian flights. The picture is indeed complex and intricate and more research is required for it to be fully untangled.

In this light, new public health policies aimed at making larger portions of the population aware of the risks of an unscheduled lifestyle, together with the discovery of new drugs that may interact with the clock molecular machinery, might prove helpful in coping with the new growing pandemic in metabolic pathologies. In addition, approaches like light therapy and chronotherapy may result useful in such a perspective.
